# Auditory Outcomes in Adolescents with Prenatal Alcohol Exposure

**DOI:** 10.1159/000528846

**Published:** 2022-12-29

**Authors:** Humberto de Oliveira Simões, Eduardo Tanaka Massuda, Erikson Felipe Furtado, Sthella Zanchetta

**Affiliations:** ^a^Department of Neuroscience and Behavioral Sciences, Ribeirão Preto Medical School, University of São Paulo, Ribeirão Preto, Brazil; ^b^Department of Ophthalmology, Otorhinolaryngology and Head and Neck Surgery, Ribeirão Preto Medical School, University of São Paulo, Ribeirão Preto, Brazil; ^c^Department of Health Sciences, Ribeirão Preto Medical School, University of São Paulo, Ribeirão Preto, Brazil

**Keywords:** Prenatal alcohol exposure, Hearing, Adolescents, Auditory perception, Auditory processing

## Abstract

The aim of the study was to investigate three aspects of auditory function (auditory acuity, cochlear dysfunction, and auditory processing) in adolescents with fetal alcohol exposure without phenotypic changes. Fifty-one adolescents with and without intrauterine exposure to alcohol were selected from a cohort study. The summons, evaluation, and analysis of the results were carried out blindly regarding the respective exposure to alcohol. The auditory tests were pure-tone audiometry, transient otoacoustic emissions, and behavioral assessment of auditory processing (speech-in-noise, dichotic digits, and gap-in-noise). After testing, 45 adolescents were included in the evaluation and were divided into exposed (*n* = 22) and non-exposed (*n* = 23) groups. Hearing loss was identified in one subject in the exposed group (4.5%). In the absence of hearing loss, there were no significant differences in tonal thresholds or in the magnitudes of the sensory (cochlear) responses between groups (*p* > 0.05). There was also no difference between the two groups regarding performance on the processing tests (speech-in-noise *p* = 0.71, dichotic *p* = 0.94, and gap-in-noise *p* = 0.33). However, the exposed group had more cases of hearing disorders (hearing loss plus auditory processing disorders) than the non-exposed group (22.7% vs. 4.3%).

## Introduction

Approximately that 9.8% of women worldwide consume alcohol during pregnancy [1]. Teratogenic effects in children who are exposed to alcohol during pregnancy range from growth deficits and facial dysmorphisms to central nervous system abnormalities, with overlapping factors and even comorbidities [1, 2, 3, 4, 5]. Because of the great variability in manifestations and levels of severity, the term “fetal alcohol spectrum disorder” (FASD) has been proposed to identify any effects resulting from this type of exposure [2, 6]. This term includes the diagnosis of fetal alcohol syndrome (FAS), partial FAS (pFAS), alcohol-related neurodevelopmental disorder, and alcohol-related birth defects [4].

Research on the conditions of the peripheral auditory system in children with FAS describes the occurrence of hearing loss and peripheral impairment more frequently, conductive hearing loss, which affects the outer and/or middle ear [7, 8, 9]. These hearing losses are estimated to affect approximately 70% of children with FAS [7, 8, 9, 10]. However, it should be noted that the research that presented these numbers studied a population with a diagnosis of FAS as well as craniofacial deformities, including cleft palate, which are characteristics that regardless of etiology are risk factors for this type of hearing loss [11]. In comparison, sensorineural hearing loss, which involves the inner ear and/or auditory nerve, was found to affect between 27% and 29% of a population with FAS [12]. The literature attributes the occurrence of hearing loss in this population to the death of hair cells in the inner ear during the embryonic phase, which reduces the number of neural progenitor cells [13, 14].

In the absence of hearing loss, the inner ear encodes acoustic parameters into electrical stimuli that are conducted and analyzed by subcortical and cortical neural structures; the efficiency with which this step occurs is called auditory processing [15, 16]. Several disorders could interrupt or make this process ineffective, called auditory processing disorders (APDs). Some of these disorders involve structural and functional alterations in the central nervous system [1, 2, 16]. However, APD can also be observed without these conditions [1, 2, 17]. Manifestations of APD include difficulties in understanding spoken messages in the presence of noise, locating a sound source, carrying out orders, and poor academic performance, among others [18]. Two studies have investigated the mechanisms of auditory processing in people with FAS [12, 19]. The first study administered speech-in-noise (SIN) and competitive attention tests in 12 of the 25 participants and found 100% altered results [12]. The second study used the dichotic verbal test and found that school-aged with a FAS diagnosis had a lower occurrence of correct stimuli from the right ear (RE) compared to the left [19]. For example, in this case, they did not have the advantage of the RE, a result expected in right-handed subjects, such as in the population studied [19]. In a dichotic test, the higher score of the RE in relation to the left ear (LE) (in free attention), known as the advantage of the RE in right-handed subjects, was described [20] as a biomarker of the left hemisphere and specificity in the processing of linguistic information. This interpretation was later corroborated by other studies, including imaging studies [21]. There is also documentation of the impact of fetal alcohol exposure on auditory processing through the electrophysiological recording of components of cortical origin, such as M1 and M2 on magneto-electroencephalography in children with FASD [22] and P2 and P3a on event-related potentials in exposed children [23].

Assuming that a major part of studies evaluated children with FAS who had morphological alterations, we proposed to investigate the auditory sensitivity, magnitude of cochlear sensory response, and responses to psychoacoustic tests that can be used to evaluate auditory processing in adolescents with fetal alcohol exposure. This study aimed to understand the auditory profile of children exposure who did not have phenotypic changes of FAS or pFAS.

## Materials and Methods

In this cross-sectional observational study, the comparative group was a cohort of adolescents who had been followed longitudinally since birth; outcome variables were evaluated at school age, 12–14 years old. The study was approved by the Human Ethics Committee of the Clinical Hospital of Ribeirão Preto Medical School − University of São Paulo (Number: 5005/2014). All children provided written informed consent, and their parents or guardians also consented and signed the consent form. The study was conducted in accordance with the World Medical Association Declaration of Helsinki.

Between 2001 and 2002, 449 women at 31–32 weeks' gestation were interviewed to characterize their consumption of alcohol during the gestational period as part of a project called Gesta-Álcool. The research tool was a structured questionnaire that included the question: “did you drink alcohol during pregnancy?”. If the answer was positive, was characterized the gestational period in which the woman consumed alcohol (first, second, or third trimester), as well as the frequency, days, and doses of consumption through the following three questions: (1) “how many days did you consume alcohol?”; (b) “what were the average doses you consumed on those occasions?”; and c) “how many times have you consumed three or more doses of alcohol?”.

### Contextualization of the Sampling

In 2013 and 2014, of the initial 449 mothers, only 81 could be located to participate in a new phase of the study called Infanto-Álcool I. This phase focused specifically on the audiological evaluation of the present research (Infanto-Álcool II). Although 75 relatives of the 81 mothers were successfully contacted, 6 mothers declined the invitation to participate and 18, despite accepting the invitation, did not show up on the evaluation day even after three attempts; finally, 51 children were evaluated.

### Sampling

The 51 children (13–14 years) included both sexes and comprised those who were exposed or not exposed to alcohol during the gestational period, and they underwent the audiological evaluation. Two audiologists with diagnostic expertise (H.O.S. and S.Z.), who were blinded to the alcohol exposure status of each child, performed the tests. The coordinator of the projects identified the children who were exposed to alcohol after the audiological assessment and then divided all children into two groups, namely, the exposed group (EG) with 22 children and the non-EG (NEG) with 23 children. Six children were excluded due to incomplete questionnaires (exclusion criterion). This blind procedure aimed to reduce the occurrence of bias.

### Characterization of Alcohol Consumption

Of the 45 children that were included in the present study, 48.9% (22/45) were children of mothers who reported alcohol ingestion at some point during pregnancy. Regarding the frequency of ingestion, 36.4% (8/22) were children of mothers who reported alcohol consumption in the first trimester, 18.9% (4/22) of the children's mothers ingested alcohol in two trimesters, and 45.4% (10/22) of the children's mothers ingested alcohol in three trimesters. In the first trimester, the duration of substance use ranged from 1 to 12 days (mean, 1.9 ± 2.4 days), the number of doses was ranged from 1 to 5 (mean, 1.7 ± 1.0), and the number of times when more than three doses were consumed ranged from 1 to 12 times (mean, 1.8 ± 2.3). In the second trimester, the duration of substance use varied from 1 to 2 days (mean, 1.2 ± 0.5 days), the number of doses was 1–5 (mean, 1.6 ± 1.2), and the number of times when more than three doses were consumed ranged from 1 to 2 times (mean, 1.2 ± 0.5). In the third trimester, the duration of alcohol consumption ranged from 0 to 1 day (mean, 0.1 ± 0.3 days), the number of doses ranged from 0 to 3 (mean, 0.2 ± 0.7), and the number of times when more than three doses were consumed ranged from 0 to 1 (mean 0.1 ± 0.3).

### Assessments

None of the children in this research had phenotypes associated with fetal alcohol exposure at 12 years of age when they were assessment by medical team that was trained by the project coordinator [24, 25]. We must acknowledge the limitation of our assessment approach because these diagnostic criteria have a low specificity in detecting alcohol exposure [26]. All the children were evaluated by a psychiatrist under the supervision of one of the authors (E.F.F.) to identify any mental disorder using the Kiddie Schedule for Affective Disorders and Schizophrenia for School-Age Children − Present and Lifetime Version (K-SADS-PL) [27, 28, 29].

### Auditory Measurements

#### Pure-Tone Audiometry

Auditory thresholds for pure tones were examined using a model MADSEN Astera^2^ (Otometrics) with headphones (TDH 39). The air-conduction threshold measurements were 0.25, 0.5, 1, 2, 3, 4, 6, and 8 kHz, and the ascending-descending procedure was used with the “up 5 dB–down 10 dB” technique. Bone-conduction threshold measurements were performed only in the presence of changes in air-conduction thresholds at frequencies between 0.5 and 4 kHz. The audiometric measurement shows a loss in dB compared with the normal hearing level (≤20 decibels hearing level [dB HL]), and the average tone loss is calculated using the proposal of the Bureau International d'Audiophonologie [30]. The speech recognition threshold was used to confirm the auditory threshold.

#### Acoustic Immittance Measures

The functional evaluation of the middle ear was performed with MADSEN Zodiac 901 middle ear analyzer model (Otometrics). We analyzed the presence of the contralateral acoustic reflex at 1 kHz and 2 kHz, with thresholds up to 110 dB HL. In addition, analysis of the tympanometry testing was performed with a 226Hz probe, and the results were classified according to Jerger [31] − type A (As and Ad) and C curves, provided that there was a contralateral reflex at 1 and 2 kHz.

#### Cochlear Function

The transient evoked otoacoustic emission (TEOAE) test was carried out with a clinical OAE analysis using Otodynamics equipment (ILO V6 model). Nonlinear clicks were used as sound stimuli, presented at a rate of 80/s and an intensity of 80 ± 3 decibels sound pressure level (dB SPL). The noise rejection was 54.9 dB equivalent in SPL, with 260 scans and a time window of 12.5 ms record. The criteria used to interpret a result as present TOAE, i.e., sensory response preserved, were ≥50% reproducibility and signal/noise ratio ≥ 3 dB in three of the four frequency bands surveyed (1.6, 2.4, 3.2, and 4 kHz), for each ear.

#### Assessment of Auditory Processing

The auditory tests used included SIN [32], dichotic digits (DDT) [32], and the gap-in-noise (GIN) [33]. These tests evaluate the mechanisms for listening to speech perception in low redundancy, dichotic verbal listening, and temporal processing conditions, which make up auditory processing, as recommended in previous studies [34]. It should be noted that the respective tests also meet another recommendation of the use of verbal and non-verbal stimuli [15].

The SIN test, a verbal test developed for the Brazilian Portuguese language, is characterized by a monaural test. Each ear is tested twice; the first test includes words that are spoken quietly (quiet condition), and the second is speech with white noise (noise condition). Twenty-five monosyllables were presented at a time at an intensity of 40 dBSL. In the noise condition, the relationship with the speech signal was +5 dB. Adequate values were considered to be a percentage of correct answers ≥70% correct answers in the noise condition, and a difference of <20% between the two conditions for the same ear, according to the authors of the test.

#### The DDT

Brazilian Portuguese is composed of the numbers 4, 5, 7, 8, and 9 distributed in 20 sequences; each sequence comprises four of these numbers, without repetition of a number in the same sequence. In each sequence, two numbers are first presented, one in each ear, and then the other two numbers are presented, one in each ear. Each pair of numbers is presented simultaneously and coincides with the initial time alignment. The test was performed at 50 dBSL. The subject was instructed to pay attention and repeat the four numbers at the end of each sequence that was heard in both ears (binaural integration). The order of the numbers when repeated was not an analysis criterion. According to the normality criteria established by the authors of the test, correct answers ≥95.0% for each ear were considered to be an appropriate result for the age of the participants in this study.

The GIN test is a temporal resolution non-verbal test. It was presented at an intensity of 50 dBSL (one track per ear). It is composed of a series of segments of 6 s of broad-band noise sequences, white noise, and an interval between sequences of 5 s. The gap durations are 2, 3, 4, 5, 6, 8, 10, 12, 15, and 20 ms, and each time interval occurs six time for each gap duration within each list, with a random distribution. The subject was told that when identifying each gap interval, it was necessary to press a button. The temporal resolution threshold, defined as the shortest gap duration identified, was considered to be ≤6 ms in each ear, according to studies of the Brazilian population [35].

DDT and GIN were used because they are only influenced moderately supramodal factors that affected auditory processing tests [18]. However, the SIN test is one of the most commonly used tests for assessing speech perception in low redundancy. Each test was interpreted as a typical or altered score depending on age. APD was identified when there were at least two tests with altered results, as recommended [15].

### Statistical Analysis

Sociodemographic characteristics and sex were examined using the χ^2^ test. Comparative analyses between groups were performed using the Mann-Whitney U test or an independent sample *t* test. For within-group comparisons between the RE and LE, the Wilcoxon test or a paired sample *t* test was used. *p* values ≤0.05 were considered statistically significant, and data were analyzed using the Statistical Package for Social Sciences (IBM SPSS 20.0, 2014) and GraphPad Prism version 7.02.

## Results

### Sample Characterization

Of the 51 participants, 88.2% (45/51) attended the hearing assessment and all participants completed the battery of tests. The ages of the adolescents ranged between 13 and 14 years old; 57.8% (26/45) were male, and 42.2% (19/45) were female. Information on the sociodemographic conditions of the mothers of the 45 subjects at birth is shown in Table 1.

None of the 22 subjects in the EG had a phenotype commonly associated with FAS or pFAS. All 45 adolescents had a psychiatric evaluation that made it possible to identify that both groups had members with some type of psychiatric disorder (37.7%, 17/45), and 41.1% (7/17) had more than one diagnosis. The most common disorder in both groups was attention deficit hyperactivity disorder (ADHD) in both groups, and the proportion of adolescents with ADHD was comparable between groups (*p* = 0.654) (Table 1). As for other psychiatric disorders, there was no significant difference between the occurrence of these disorders according to group (social phobia *p* = 0.598; oppositional defiant disorder *p* = 0.273; and specific phobia, enuresis, and encopresis *p* = 0.623).

### Auditory Sensitivity

Only one subject (4.5%, 1/22 in the EG) had altered thresholds with very severe mixed first-degree hearing loss, i.e., unilateral hearing loss in the LE. After otorhinolaryngological evaluation, the patient was diagnosed with labyrinthitis ossificans and was excluded from the other evaluation and analysis steps.

**Fig. 1:**
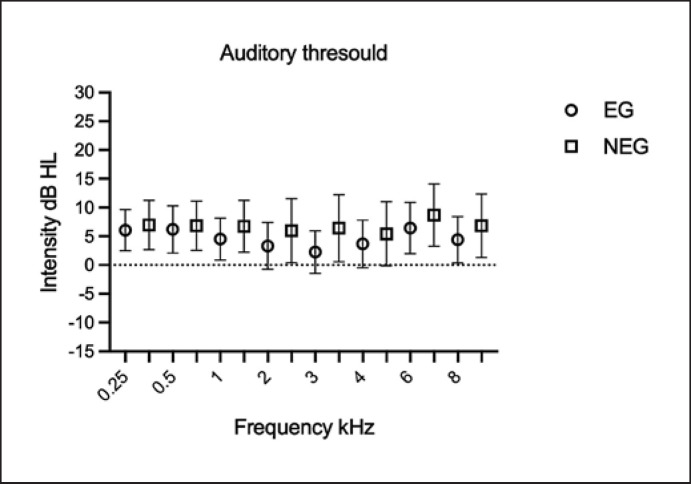
Mean value comparison of air-conducted thresholds between groups (*n* = 44 adolescents).

The remaining 44 subjects had hearing thresholds ≤20 dB HL. The comparative study of the airway thresholds between the RE and LE in each group showed that there was no difference for any frequency (EG: 0.25 kHz, *p* = 1.0; 0.5 kHz, *p* = 1.0; 1 kHz, *p* = 1.0; 2 kHz, *p* = 0.387; 3 kHz, *p* = 0.113; 4 kHz, *p* = 1.0; 6 kHz, *p* = 0.381; and 8 kHz, *p* = 0.826; NEG: 0.25 kHz, *p* = 0.613; 0.5 kHz, *p* = 0.171; 1 kHz, *p* = 0.307; 2 kHz, *p* = 1.0; 3 kHz, *p* = 1.0; 4 kHz, *p* = 1.0; 6 kHz, *p* = 0.744; and 8 kHz, *p* = 0.627). Figure 1 shows the audiometric pattern, by ear; statistical non-significance allows the groups to be combined.

### Cochlear Function

**Fig. 2:**
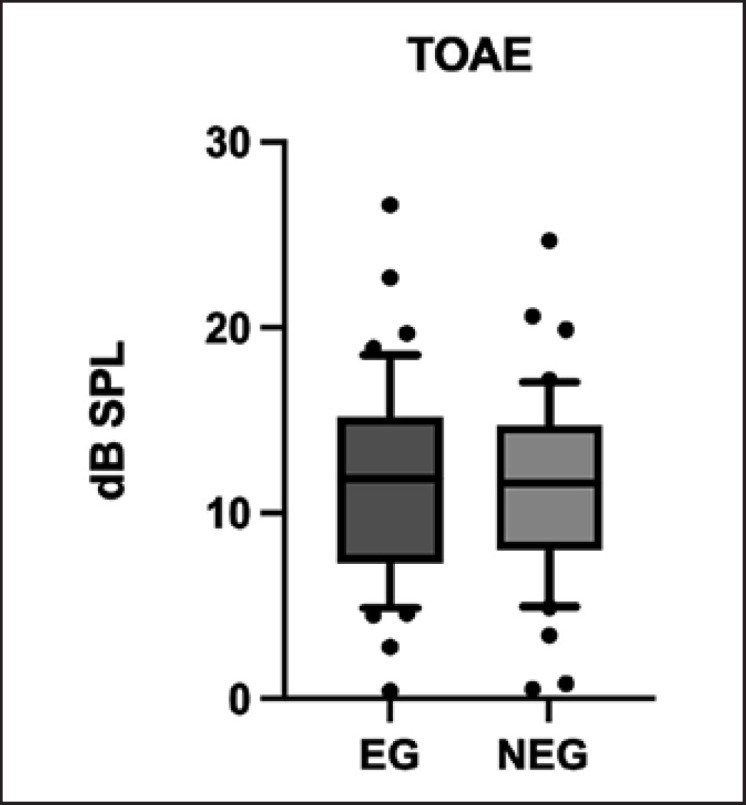
Average and 95.0% confidence interval of the TOAE amplitude response.

The TEOAE examination was performed for all 44 subjects with sensitivity within normal standards, and the presence of responses was observed in both ears. First, the magnitude of the responses between the RE and LE was analyzed (considering the groups separately), and there was no difference in the amplitude values (EG: *t* = −0.12 and *p* = 0.781; NEG: *t* = 0.63 and *p* = 0.302). This outcome allowed the results of the two ears to be combined for comparative analysis between the two groups. In the NEG, 23 subjects (46 ears) were included, and in the EG, 21 subjects (42 ears) were included. The ± of the response (dB SPL) was 11.22 ± 5.52 dB SPL for the NEG and 11.54 ± 4.93 dB SPL for the EG. The results of the independent sample *t* test showed no difference between them (*t* = 0.28; *p* = 0.776) (Fig. 2).

### Assessment of Auditory Processing

Only subjects with adequate auditory sensitivity performed the auditory processing assessment (*n* = 44). The results of the three tests showed the presence of altered scores in both groups. The joint analysis allowed us to identify central APD (CAPD) in 4.3% (1/23) of the children in the NEG and in 14.3% (3/21) in the EG (Fisher's test *p* = 0.186), with a relative risk of 1.7 (95% confidence interval [CI] 0.789–2.834). When analyzing scores according to a test, in consideration of normal versus altered results, we found the following: in the SIN test, altered results occurred in 13.0% (3/23) of children in the NEG and in 9.5% (2/21) of those in the EG; two children (one in each group) had abnormal TDD results (EG: 4.7%; NEG: 4.3%). Regarding the GIN test, only one subject belonging to the NEG had abnormal results (4.3%, 1/23). We compared the results (by percentage) of the tests between groups according to ear, and no significant difference was found for any of the tests (*p* > 0.05, Table 2).

In the SIN test for the quiet condition, the median for the RE was 93.5% for the NEG and 94.1% for the EG (95% CI, 92.0–100.0% NEG vs. 88.0–100.0% EG, U' = 211.5; *p* = 0.438); for the LE, it was 93.5% for the NEG and 94.1% for the EG (95% CI, 88.8–100.0 for NEG and EG, U' = 225.5; *p* = 0.720). In the noise condition, the EG presented with lower values, but the difference was not significant (*p* > 0.05). The median values for the RE were 80.3% and 78.2% for the NEG and EG, respectively (95% CI, NEG 72.8–88.0 vs. EG 64.8–88.0, U' = 193.5, *p* = 0.245); for the LE, the median was 79.3% for both groups (95% CI, NEG 68.8–91.2 vs. EG 58.0–87.6, U' = 200.0, *p* = 0.320).

The results of the DD test were homogeneous between the two groups, with no difference, for both ears (*p* > 0.05). The median values for the RE were 98.5 for both groups (95% CI, NEG 94.2–100.0 vs. EG 93.0–100.0, U' = 193.5, *p* = 0.245); for the LE, the medians were 98.7 for the NEG and 97.5 for the EG (95% CI, NEG 96.2–100.0 vs. EG 91.8–100.0, U' = 184.5, *p* = 0.165).

For the GIN test, the results also showed no differences between the NEG and EG for both the RE and LE (*p* > 0.05). The median temporal resolution threshold was 5 ms for both ears in both groups (95% CI, RE, NEG 3.2–6 vs. EG 4–6, U' = 213.0; *p* = 0.488; LE, NEG 3.2–7.6 vs. EG 4–6, U' = 241.5; *p* = 1.00).

The presence or absence of hearing disorders (hearing loss plus APD) was 22.7% and 4.3% of the participants in the EG and NEG, respectively. Although not significant, the results of Fisher's test tended toward significance (*p* = 0.096) and revealed a relative risk of 1.9 for EG to NEG (95% CI, 0.936–3.026).

Upon examination of a potential relationship between the fetal alcohol exposure factor and the most frequent psychiatric disorder in the sample, ADHD, as well as CAPD, the exact χ^2^ test showed no association between alcohol consumption and the disorders (ADHD, *p* = 0.72; and CAPD, *p* = 1.00). The logistic regression between ADHD (*p* = 0.90; odds ratio [OR] 1.2; 95% CI, 0.069–20.854) and CAPD (*p* = 0.59; OR 1.5 and 95% CI: 0.341–6.607) did not show statistical significance.

## Discussion

The present study investigated the auditory profiles, auditory sensitivity, magnitude of the cochlear sensory response, and auditory behavioral processing of adolescents with fetal exposure to alcohol, but without the phenotypic characteristics commonly associated with the condition. To the best of our knowledge, no previous study has been conducted with the characteristics of the present sample in which auditory function has investigated a set of tests. Differences in the characteristics of study populations are essential and should be considered in the following topics.

### Sample Characterization

For the constitution of the groups, the results of the SQ were adopted because it aims to track the consumption pattern of pregnant women with a “yes” or “no” answer, with the identification of the gestational period. The SQ identified that 48.9% of pregnant women in the present sample consumed some amount of alcohol during pregnancy. This percentage is slightly higher than the estimate in Brazil, which is 20.0–40.6% [36, 37]. In terms of sociodemographic characteristics of the mothers between the two groups, no significant differences were found.

The follow-up of the sample over several years allowed for finding that none of the children had presented, in their development, the set of phenotypic signs traditionally related to fetal alcohol exposure. This characteristic is important for the discussion of the results of auditory tests performed here, as it can be inferred that they presented with less severe effects of alcohol [3, 4]. All adolescents underwent a psychiatric evaluation during “Infanto-Álcool I.”

Among psychiatric diagnoses, attention should be paid to the high occurrence of ADHD, in both the groups. ADHD has an estimated prevalence 2–7% in the infant population [38], but it has been identified in 94.8% of those with FAS using K-SADS [39]. The fact that the participants in the present study did not have signs of FAS may justify the higher occurrence of ADHD than in the general population, but it is lower than that reported in the study by Fryer et al. [39]. Regarding the unexposed group, the occurrence was higher than that in the general population [38], and it was higher than the 30% reported by Fryer et al. [39]. It is necessary to note the possibility of the occurrence of two sources of bias. First, the children who participated in our study already had behaviors that were identified as suspicious by their parents, which led to their inclusion in the study 12 years after the first phase. Second, there are reports regarding the relationship between low socioeconomic status and ADHD [40, 41]. The authors suggest that a lower socioeconomic level increases the risk of ADHD; however, there is a more significant occurrence of psychiatric disorders among family members, people who consume more drugs and tobacco, etc. [41]. There was no difference in family income between the groups, but more than half of the respective families could be classified as low-income families in Brazil (up to five minimum wages [MWs]).

### Auditory Sensitivity

The prevalence rate of hearing loss identified in the EG was 4.5%; however, this is lower than that reported in the literature on any hearing loss in children exposed to alcohol during pregnancy, which ranges from 70 to 77% [7, 8, 9], and for sensorineural hearing loss, which is estimated to be 29% [7, 8, 9, 12]. It is necessary to consider the sample composition of the subjects studied here, who had fetal alcohol exposure, but did not have the phenotypic alterations commonly associated with FAS, possibly indicating lower exposure severity. The high frequency of conductive losses owing to external and middle ear alterations has been described in a population with FAS and craniofacial deformities, including, but not limited to, cleft palate, and characteristics that are independent of the conductive hearing loss etiology are risk factors for this type of loss [7, 8, 9, 11, 12]. Sensorineural hearing loss was previously reported to be between 27% and 29% in the presence of FAS [12]. Studies have reported that fetal alcohol exposure can damage the optical placoid of guinea pigs as well as cause deformities in the stereocilia of hair cells and sustentation; fetal alcohol exposure can also cause embryological damage to the inner ear and cranial nerve VIII [8, 13, 14, 42, 43].

However, hearing loss of one adolescent in the EG in this study was identified to be of a mixed type (middle and inner ear impairment), and this is not necessarily representative of the FAS population [7, 8, 9, 12]. The otorhinolaryngological diagnosis for this mixed hearing loss was labyrinthitis ossificans, which is characterized by ossification of the membranous labyrinth, is usually related to a sequel of otological infection, often suppurative labyrinthitis or meningitis, and is not related to ingestion of alcohol during pregnancy [44, 45, 46].

Another result that is contrary to previous reports is that for children without hearing loss in both groups, the thresholds for pure tone were similar. Children with FAS without hearing loss tended to have lower thresholds for frequencies from 3 to 8 kHz (on average 5 dB HL), whereas for frequencies from 9 to 20 kHz, the decrease was greater, from 3 to 14 dB HL [47]. Unfortunately, pure-tone audiometry at high frequencies (>8 kHz) was not possible in the population studied here.

### Cochlear Function

Although otoacoustic emission is not an audibility test, the recording of the contractility of outer hair cells, as a result of a sound, provides relevant information on cochlear sensory function [48]. No differences were found in these responses between adolescents with and without fetal alcohol exposure, as has already been reported [47]. Katbamna et al. [47] evaluated eight subjects with FAS in the age range of 8–17 years using distortion product otoacoustic emissions, which indicated a tendency of decreased amplitude in the frequency range of 4–8 kHz. The divergence from the results of the cochlear response pointed out above can be attributed to the type of otoacoustic emissions used. The TEOAE (chosen by us) does not provide the specificity of sound frequency, as that of the respective author mentioned above; they used the distortion product otoacoustic emission method. However, it can also be suggested that the adolescents in this study experienced less severe fetal alcohol exposure, which is compatible with the absence of phenotypic signals.

### Assessment of Auditory Processing

The two groups studied showed results suggestive of alteration in at least one auditory processing test, but there was no statistical difference between them. Our results differ from those of other reports [12, 19]. There are some hypotheses for this divergence, such as the differential diagnosis regarding the severity of exposure to alcohol and the occurrence of morbidities in the NEG. The samples evaluated in other studies required a diagnosis of FAS, whereas the adolescents in the present study had not been diagnosed with FAS diagnosis. The second hypothesis concerns the presence of comorbidities in the NEG. The occurrence of psychiatric disorders was the same between the two groups, including ADHD, which is a well-documented condition identified in APD [34, 47]. Thus, both the groups presented risk criteria for altered results of their respective tests [15]. Regardless of whether they were exposed to alcohol during pregnancy, it is still possible to record the overlap between these disorders since children diagnosed with APD also have symptoms of inattention and hyperactivity [18, 49]. The justification for the overlap is the organization of specific neural networks that are shared for more than one activity [50]. However, some studies suggest that APD is more likely to overlap with language impairment and reading disorders than with ADHD [51, 52]. The third and final hypothesis is related to differences in the type of test used and the peripheral hearing condition of the adolescents evaluated between the studies. Church et al. [10] used two tests, SIN and competitive sentences in contralateral (dichotic) modality, and reported that 100% of the subjects had altered results for at least one of the two tests. However, it is important to consider that of the 22 subjects who were recruited, 17 had peripheral hearing loss when auditory processing was evaluated. Of these 22 subjects, 12 underwent behavioral tests. Peripheral hearing loss is not an impediment to auditory processing, but it negatively influences performance [34]. Thus, although the authors mentioned above described a high percentage of altered results, it is not possible to distinguish between acoustic (peripheral) encoding damage and neural processing as the reason for those results. Domellöf et al. (2009) [19] used only one behavioral auditory test, the verbal dichotic with words, in the stages of directed listening. In a dichotic stimulation, if only one of the two stimuli is repeated, those coming from the RE will be the most frequent in right-handed subjects. This performance would reveal the dominance of the left hemisphere for processing verbal language in right-handed subjects [20]. In contrast, ignoring stimuli from the RE and repeating only the stimuli from the LE would imply that the performance of the “top-down” mechanism that is cortical, non-auditory modalities would be acting on the auditory performance [34]. This mechanism was the objective of the study, and the authors showed that right-handed children with FAS had worse directed listening performance than children in a compared to a control group. However, the authors attributed this to developmental disorders or focal brain injury [19]. We also used a dichotic verbal test; however, it is not comparable to the tests used by the authors because we did not perform targeted listening, since we aim to have tests with less influence of top-down mechanisms on auditory tests.

### Final Considerations

Despite measures taken to avoid bias, it is essential to note its occurrence. Longitudinal design of the research is a bias. Although the study design allowed the identification of alcohol consumption during pregnancy, which gave more reliability to the report, some participants in the initial sample were lost. The second bias was related to the unexposed group and the high occurrence of ADHD, as mentioned and discussed earlier. Given that FASD is characterized by numerous manifestations, many of them without phenotypic or behavioral markers, there is a need to identify protocols that are sensitive to the identification of the condition. As far as we know, no other study has mapped the auditory function in a population exposed to alcohol during pregnancy, without the typical dysmorphological manifestations. The children studied here have a higher risk of auditory disorders (hearing loss plus APD) when compared to those not exposed to alcohol. There is a need for further studies from the point of view of diagnosis that encompasses high-frequency audiometry, the efferent auditory system, and the inclusion of temporal ordering auditory processing tests to elucidate other aspects of the auditory function not contemplated until now.

## Conclusion

Adolescents with fetal alcohol exposure who did not have phenotypic changes of FAS or pFAS had more hearing disorders than the NEG. APD was more prevalent in the EG than in the NEG; however, there were no significant differences. The lack of contrast in the results can be attributed to the high occurrence of psychiatric disorders in both groups.

## Statement of Ethics

The study was approved by the Human Ethics Committee of the Clinical Hospital of Ribeirão Preto Medical School − University of São Paulo (Number: 5005/2014). All children provided written informed consent, and their parents or guardians also consented and signed the consent form. The study was conducted in accordance with the World Medical Association Declaration of Helsinki.

## Conflict of Interest Statement

The authors have no conflicts of interest to declare.

## Funding Sources

This research was supported by Grant No 162123/2014-0 from the CNPq (Brazil), awarded to Simões for postgraduate (master's degree).

## Author Contributions

Dr. Furtado had full access to all the data from the “Infanto-Álcool I” and “Gesta-Álcool” thematic project. Study concept: Simões and Zanchetta. Data acquisition: Simões. Distribution of participants in groups: Furtado. Medical care support to participants: Massuda. Data Analysis or interpretation and drafting of the manuscript: Simões, Zanchetta, and Massuda.

## Data Availability Statement

All data generated or analyzed during this study are included in this article. Further inquiries can be directed to the corresponding author.

## Figures and Tables

**Table 1:** Sociodemographic characteristics of mother and psychiatric diagnosis of the sample due to fetal alcohol exposure (*n* = 45)

Variables	Categories	Total (*N* = 45)	NEG (*N* = 23)	EG (*N* = 22)	*X*^2^; *p* value	OR
Child sex	Male	26 (57.8)	15 (65.2)	11 (50.0)	1.06; 0.30	−
	Female	19 (42.2)	8 (34.8)	11 (50.0)		

Skin color	White	27 (60.0)	11 (47.8)	13 (59.1)	2.56; 0.27	−
	Black	10 (22.2)	9 (39.1)	4 (18.2)		
	Brownish	8 (17.8)	3 (13.1)	5 (22.7)		

Marital status	Not married	17 (37.8)	3 (13.1)	2 (9.1)	0.28; 0.86	−
	Married	28 (62.2)	20 (86.9)	20 (90.9)		

Educational level	Basic education	10 (22.2)	4 (17.4)	6 (27.3)	1.43; 0.48	−
	Elementary school	19 (42.2)	9 (39.1)	10 (45.4)		
	High school	16 (35.6)	10 (43.5)	6 (27.3)		

Maternal employment	Active	16 (35.6)	10 (43.5)	6 (27.3)	1.45; 0.48	−
	Inactive	19 (42.2)	8 (34.8)	11 (50.0)		
	Withdrawal	10 (22.2)	5 (21.7)	5 (22.7)		

Family income	Up to 1 MW	1 (2.2)	1 (4.4)	0 (0.0)	2.26; 0.51	−
	From 1 to 5 MW	27	13 (56.5)	14 (63.6)		
	From 6 to 10 MW	17	9 (39.1)	7 (31.9)		
	More than 10 MW	1 (2.2)	0 (0.0)	1 (4.5)		

Religion	Catholic	35 (77.8)	17 (73.9)	18 (81.9)	0.54; 0.91	−
	Others	8 (17.8)	5 (21.7)	3 (13.6)		
	Without religion	2 (4.4)	1 (4.4)	1 (4.5)		

Child psychiatric diagnosis	ADHD	9 (20.0)	4 (44.4)	5 (55.6)	0.200; 0.654	1.39
	Social phobia	5 (11.1)	2 (40.0)	3 (60.0)	0.277; 0.598	1.65
	ODD	4 (8.8)	1 (25.0)	3 (75.0)	1.198; 0.273	3.47
	Specific phobia	3 (6.6)	1 (33.4)	2 (66.6)	0.406; 0.623	2.20
	Enuresis	3 (6.6)	1 (33.4)	2 (66.6)	0.406; 0.623	2.20
	Encopresis	3 (6.6)	1 (33.4)	2 (66.6)	0.406; 0.623	2.20
	PTD	1 (2.2)	1 (100.0)	0 (0.0)	−	−
	SAD	1 (2.2)	0 (0.0)	1 (100.0)	−	−
	OCD	1 (2.2)	0 (0.0)	1 (100.0)	−	−

MW, minimum wage; Brazilian currency, Real (R$); NEG, non-exposed group; EG, exposed group; ADHD, attention deficit hyperactive disorder; ODD, oppositional defiant disorder; PTD, provisional tic disorder; SAD, separation anxiety disorder; OCD, obsessive-compulsive disorder; OR, odds ratio; *n,* sample number; %, percentage.

**Table 2:** Descriptive analysis of the auditory processing behavioral assessment results

Auditory behavior tests	Ear	NEG, *n* = 23		EG, *n* = 21	
		median	min-max	median	min-max
SIN^1^ (%)	RE	92	92–100	96	88–100
	LE	92	88–100	96	88–100

SIN^2^ (%)	RE	80	72–88	80	64–88
	LE	80	68–88	80	56–88

DD (%)	RE	98.7	93.7–100.0	98.7	92.5–100
	LE	98.7	96.2–100.0	97.5	91.2–100

GIN, ms	RE	5	3–6	5	4–6
	LE	5	3–8	5	4–6

NEG, non-exposed group; EG, exposed group; RE, right ear; LE, left ear; quiet condition; noise condition; listening condition with white noise; DD, dichotic digits test; GIN, gaps-in-noise test.

1 SIN, speech-in-noise test.

2 SIN, speech-in-noise test.
